# Trends in Socioeconomic Inequalities in Norwegian Adolescents’ Mental Health From 2014 to 2018: A Repeated Cross-Sectional Study

**DOI:** 10.3389/fpsyg.2020.01472

**Published:** 2020-07-07

**Authors:** Arnhild Myhr, Kirsti S. Anthun, Monica Lillefjell, Erik R. Sund

**Affiliations:** ^1^Department of Neuromedicine and Movement Science, Faculty of Medicine and Health Sciences, Norwegian University of Science and Technology, Trondheim, Norway; ^2^Trøndelag R&D Institute, Steinkjer, Norway; ^3^Department of Public Health and Nursing, Faculty of Medicine and Health Sciences, HUNT Research Centre, Norwegian University of Science and Technology, Levanger, Norway; ^4^Faculty of Nursing and Health Sciences, Nord University, Levanger, Norway; ^5^Levanger Hospital, Nord-Trøndelag Hospital Trust, Levanger, Norway

**Keywords:** trends, socioeconomic inequalities, adolescents, mental health, depression, anxiety, loneliness

## Abstract

**Background:**

Adolescents’ mental health, and its consistent relationship with their socioeconomic background, is a concern that should drive education, health, and employment policies. However, information about this relationship on a national scale is limited. We explore national overall trends and investigate possible socioeconomic disparities in adolescents’ mental health, including psychological distress and symptoms of depression, anxiety, and loneliness in Norway during the period 2014–2018.

**Methods:**

The present study builds on data retrieved from five waves of the national cross-sectional Ungdata survey (2014–2018). In total 136,525 upper secondary school students (52% girls) completed the questionnaire during the study period. Trends in socioeconomic inequalities were assessed using the Slope Index of Inequality (SII) and the Relative Index of Inequality (RII).

**Results:**

The prevalence of students with moderate to high symptoms score and mean symptoms scores of psychological distress (in terms of symptoms of depression, anxiety, and loneliness) increased among girls and boys during 2014–2018, with girls showing higher rates. Our results suggest distinct, but stable, inequalities between socioeconomic groups, both in absolute and relative terms, among girls and boys during the study period.

**Conclusion:**

Rising rates of adolescents’ psychological distress, particularly among girls, may have long-term consequences for individuals involved and the society as a whole. Future studies should investigate the causes of these results. We did not find evidence of any change in inequalities in adolescents’ mental health between socioeconomic groups, suggesting current strategies are not sufficiently addressing mental health inequalities in the adolescent population and therefore a significant need for research and public health efforts.

## Introduction

Young people growing up today are generally in good health and experience a high quality of life, especially in high-income Western countries like Norway. However, their health status at birth, risk, and resilience factors during childhood and adolescence and their future life chances are distributed and shaped according to the social gradient (Marmot, 2005; Marmot et al., 2008; Evans et al., 2012). Inequities in the socioeconomic status (SES) in which people are born, live, work, and age, driven by inequities in resources, money and power, result in inequities in health. And these inequities can also affect young people’s opportunities for education, access to health care, leisure activities, occupation, and fulfillment (Marmot, 2005; Viner et al., 2012; Hjorth et al., 2016; Moscelli et al., 2018; Sallis et al., 2018). Patterns that systemically disfavor young people with lower SES are present within and between countries, municipalities, and neighborhoods (Inchley and Currie, 2013; Barbalat and Franck, 2020).

A World Health Organization report (Inchley and Currie, 2013) documenting persistent and increasing social inequalities in a wide range of self-reported health issues and health determinants also shows growing prevalence of non-communicable diseases (NCDs), including mental health problems in the young population. Deteriorating psychological health among children and adolescents in high-income countries has been widely documented (Kosidou et al., 2010; von Soest and Wichstrøm, 2014; Potrebny et al., 2019). Moreover, studies have suggested that national income inequality is related to physical health symptom loads, lower physical activity, higher body mass index, and higher mental health symptom loads (World Health Organization [WHO], 2008; Viner et al., 2012; Elgar et al., 2015). However, the relationship between mental health problems, educational attainment, and non-participation in work/education is complex (De Ridder et al., 2012, 2013). There is a strong and bidirectional association between mental health problems and secondary school dropout (De Ridder et al., 2013; Esch et al., 2014). Non-completion of secondary school is one of the major risk factors for permanent exclusion from the labor market (OECD, 2018a,b) and low education and adverse living conditions increase the risk for developing mental health problems (Elstad, 2008). Moreover, poor mental health influences the wider health and development of adolescents and is also associated with higher alcohol, tobacco, and illicit substances use, adolescent pregnancy, and anti-social behaviors. Together, these factors may result in a downward spiral, increasing socioeconomic inequalities in health during the life course (Haas, 2008; Allen et al., 2014; Kim and Radoias, 2019).

Mental health problems, covering a wide range of illnesses, from mild or moderate anxiety and depression disorders, drug and alcohol use disorders to more severe depression, bipolar disorders and schizophrenia, are one of the leading causes of disability in children and youth (OECD, 2018b). Children and adolescents from minority groups, such as cultural/ethnic minorities or children with disabilities, are particularly vulnerable to experiencing mental health problems and challenges with participation in work, education, and leisure activities (Ytterhus et al., 2008; Viner et al., 2012; Allen et al., 2014). Several mental illnesses are more common in teenage girls and women, such as anxiety, depression, and bipolar disorders (Albert, 2015; Riecher-Rössler, 2017; Yu, 2018), while drug and alcohol use disorders are overrepresented in the male population (Foster et al., 2015).

Mental health problems account for a quarter of all years lived with disability globally (World Health Organization [WHO], 2008; Viner et al., 2012). In 2016 more than one in six (17.3%) people living in European countries suffered from a mental health problem, and the prevalence was even higher in Norway, at 18.5% (OECD, 2018b). In Norway, the prevalence of mental health complaints is increasing, especially among young people and marginalized groups (OECD, 2018b; Potrebny et al., 2019). A study by Bøe et al. (2012), of a Norwegian sample of children found that low family income predicted mental health problems. This is supported by a recent study by Zhou et al. (2018) who found that family SES had a significant impact on depressive symptoms. Moreover, national surveys of adolescents’ health in Norway show a particularly worrying trend in which girls in upper secondary school showed a sharp increase in the incidence of a high level of psychological distress, from 25.7% in 2011 to 29.3% in 2017 (Bakken, 2018). During the same time period rates of mental disorder diagnosis in girls 15–17 years climbed from 5 to 7% per year (Norwegian Institute of Public Health, 2017). Mental health problems, especially depression, have been identified as the largest cause of disability among young people in all regions in Norway (Brage and Thune, 2015). During the transition from adolescence to young adulthood, loneliness, defined as a subjective feeling of distress arising when social connections are perceived to be inadequate or unfulfilling (Matthews et al., 2016), has been identified as a specifically strong and prevalent risk factor for depression (Cacioppo et al., 2006; Qualter et al., 2010; Matthews et al., 2016). Although the prevalence of loneliness varies with age, mainly due to the fact that an individual’s social needs will shift over the course of life, its association with depression remains stable during the life course (Cacioppo et al., 2010). Children and adolescents who experience loneliness are often also at risk for anxiety and suicidality (Gallagher et al., 2014; Junker et al., 2017).

Several theories address how SES affects children’s cognitive and socioemotional development, and a general difference between them is whether they are based on social determinants (i.e., that socioeconomic conditions affect health) or social selection (i.e., that health affects SES) (Adler and Ostrove, 1999). However, these two perspectives are not mutually exclusive and affect each other within given social contexts and across generations (Conger et al., 2010; Reiss, 2013). The complex interplay between social determinants and social selection is, for example, well-described in the life course perspective, a framework considered important for understanding the development of mental health (Fryers and Brugha, 2013). A number of studies suggest that, in addition to biologic aspects, the most important mediating variables between SES and young people’s mental health are related to their parents and the family environment (Conger et al., 1995, 2010; Costello et al., 2003; Masarik and Conger, 2017; Akee et al., 2018). Two dominant perspectives for understanding the role of parents in this complex relationship are the family process model (Conger et al., 1994) and the family investment perspective (Haveman and Wolfe, 1995), which, respectively, highlight the impact of the family’s affective and structural components in children’s cognitive and socio-emotional development. Reviews of studies applying the family process model, or the family stress model (Conger et al., 2010; Masarik and Conger, 2017) display substantial empirical support for the family stress pathways and show that the model has been expanded upon and strengthened. Advancements have, for instance, articulated more fully accounts of family stress processes and included feedback loops from parents’ relationship dynamics, adult psychological functioning, and economic problems (Masarik and Conger, 2017). As a result, the model provides a more complete understanding of how economic stress influences child development across many domains through a variety of mechanisms. The family process model and the family investment perspective both assume that SES plays a major role when it comes to family functioning and the lives of family members (Conger et al., 2010). Although the family environment is considered most crucial for children’s and youth‘s development, it is reasonable to assume that more distal surroundings such as their neighborhood and school peers also strongly influence adolescents. For example, according to the relative deprivation theory (Adjaye-Gbewonyo and Kawachi, 2012; Mishra and Carleton, 2015), how disadvantaged adolescents perceive their situation relative to others is crucial to its effect on their psychological health.

Health and health behavior correlate strongly from adolescence to adulthood and thus such disparities appear to persist throughout the life course (Marmot et al., 2008). Evidence indicates that about half of all lifetime mental health problems start by mid-teens (Kessler et al., 2007). Given that adolescence is a critical stage of the life-course when patterns that determine current and future health outcomes are established (Fletcher and Sarkar, 2013), examination of the mental wellbeing and health in this population-group is vital. Moreover, analyzing trends in the populations’ health and health inequalities is a prerequisite for examining whether interventions and policy have been successful in addressing them. The aim of the present study is to explore national trends and detect possible socioeconomic inequalities in psychological distress, depressive and anxiety symptoms, and loneliness among adolescents in Norway during the period 2014–2018 and whether patterns differ by gender. We hypothesized, first, that average mental health worsened. Second, that girls’ mental health would worsen more than boys’ mental health. Third, and finally that inequalities according to socioeconomic position would widen over time. Findings, by illuminating adolescents’ mental health conditions in relation to their socioeconomic background, should drive changes in education, health, and employment policies.

## Materials and Methods

### Study Design and Participants

This study uses data collected in five waves (2014–2018) of the cross-sectional Ungdata survey (Bakken, 2016, 2018). Ungdata is a quality assured and standardized system designed to conduct local surveys among adolescents attending lower and upper secondary school in Norway (Frøyland, 2017). The survey is conducted by the Norwegian Social Research institute (NOVA) in cooperation with regional centers for drug rehabilitation (KoRus) and is financed by the Norwegian Directorate of Health. The school administrators in all Norwegian municipalities may order the survey free of charge. The students’ participation is voluntary and informed consent was obtained from the students, as well as from parents if the student was under 16 years of age. The Ungdata survey covers different aspects of young people’s lives – including school issues, leisure time activities, relationships with parents and friends, local environment, and physical and mental health – and is thus an important source of information on adolescents’ health and well-being, both at municipal and national levels. A detailed description of the content and theoretical framework of the Ungdata survey is available elsewhere (Frøyland, 2017).

In this study we included all students in upper secondary school who completed the Ungdata survey in the period 2014–2018. The response rate of the surveys varies between different surveys, schools, and school year. The overall response rate among upper secondary school students was 66 per cent across the surveys conducted in 2014–2016 (Bakken, 2016) and 69 per cent across the surveys in 2016–2018 (Bakken, 2018). In total, 144,239 students completed the Ungdata survey in the period 2014–2018. We excluded individuals with missing information on gender (*n* = 6,072), school year (*n* = 955) and family affluence (*n* = 687). The final data set contained 136,525 individuals (52% girls) who completed the Ungdata survey in 2014 (*n* = 13,345), 2015 (*n* = 25,569), 2016 (*n* = 22,752), 2017 (*n* = 37,208), and 2018 (*n* = 17,911). The sample was further reduced in the parametric estimations because of the individuals missing information on total mean scores of psychological distress (*n* = 10,136), symptoms of depression (*n* = 9,079), symptoms of anxiety (*n* = 10,083) and loneliness (*n* = 9,490).

### Measures

#### Family Affluence as a Proxy of Socioeconomic Status

We measured family SES using four items of the Family affluence scale II of material assets or common indicators of wealth (Currie et al., 1997, 2008). The four items were as follows: “Does your family have a car?” “Do you have your own bedroom?” “How many times have you traveled somewhere on holiday with your family over the past year?” and “How many computers or tablet computers does your family have?” This scale has been validated along with several other measures of adolescents’ SES. Compared to measures of SES where adolescents report their parents’ occupation, educational attainment or household income, this scale has been found to have better criterion validity and less susceptibility to non-response bias (Torsheim et al., 2004).

Since 2014 the Ungdata survey also includes other measures of adolescents’ SES, such as parental education levels, number of books in the home, and a subjective assessment of the family’s financial situation. In this study, we used a collective socioeconomic measure based on the questions about parents’ education level, the number of books in the home and the questions from FAS II, developed by Bakken et al. (2016) to reflect families’ affluence level. A mean sum score, ranging from 0 to 3, was calculated and then split into five family affluence groups of equal size (cut-off at the 20th, 40th, 60th, and 80th percentile, with the wealthiest representing the lowest group).

#### Psychological Distress, Symptoms of Depression, and Anxiety

Psychological distress was measured using the 10-item Hopkins Symptom Checklist, consisting of two subscales: a depression dimension (six items that constitute the “Depressive Mood Inventory”), an anxiety dimension (4-items, α = 0.82) and in addition to a total mean score (10-items, α = 0.93) (Derogatis et al., 1974; Strand et al., 2003).

The adolescents were asked if they had experienced each of the following symptoms during the preceding week: “Felt that everything is a struggle,” “Had sleep problems,” Felt unhappy, sad or depressed,” “Felt hopelessness about the future,” “Felt stiff or tense,” “Worried too much about things,” “Suddenly felt scared for no reason,” “Felt constant fear or anxiety,” “Been nervous or felt uneasy,” and “Felt worthless.” Each item/symptom was answered on a four-point scale ranging from “Not at all” (1) to “Very much” (4).

Separate measures for depressive and anxiety symptoms were constructed by adding up the scores (1–4) on all the items covering each dimension (10 items in total; 6 items for depression and 4 for anxiety) and dividing it by the number of completed items, given response to at least half of the statements for each scale. This resulted in two mean symptom scale scores, one for depression and one for anxiety, each ranging from 1 to 4. A total mean score “Psychological distress” was constructed by combining the depression and anxiety symptom scores. In addition to the mean score, a validated cutoff score of ≥ 1.85 was used for identifying adolescents reporting moderate to high symptom load related to overall psychological distress, depression, and anxiety symptoms (Strand et al., 2003).

#### Loneliness

The prevalence of loneliness in the adolescent population is increasing (Madsen et al., 2019) and several studies report associations with other psychological measures, in particular depressive symptoms (Cacioppo et al., 2010). Symptoms of loneliness was measured by asking the adolescents to rate, on a four-point scale ranging from “Not at all” (1) to “Very much” (4), whether they had “Felt lonely” during the last week. In addition, we constructed a dichotomous variable identifying adolescents who reported that they had felt lonely “very much” during the last week.

### Statistical Methods

Mean average symptoms scores in all mental health domains – psychological distress, depressive and anxiety symptoms and loneliness – was calculated for each gender and adjusted for the respondent’s school year and whether the respondent lives in Oslo. In addition, we calculated gender-specific prevalence of adolescents reporting moderate to high symptom loads for each of the indices.

We calculated the Relative Index of Inequality (RII) and Slope Index of Inequality (SII) in order to measure the magnitude of relative and absolute socioeconomic inequalities in the four mental health outcomes. Mackenbach and Kunst (1997) recommends using RII and SII when making comparisons across population groups or over time. Rather than comparing the two most extreme groups, these two regression-based indices take into account the entire socioeconomic distribution of the population. In this study, family affluence (proxy for SES) was transformed into a weighted summary measure scaled from 0 (highest level of affluence) to one (lowest level of affluence), to reflect the proportion of the sample represented at each affluence level. Finally, a modified ridit-score based on the midpoint of the range in the cumulative distribution of the population at a given wealth level was assigned to the population at each affluence level.

In line with recommendations in the literature (Barros and Hirakata, 2003; Spiegelman and Hertzmark, 2005), we used generalized linear models with a logarithmic link function to calculate RIIs (rate ratios) and with an identity link function to calculate SIIs (rate differences). Both SIIs and RIIs were estimated with 95% confidence intervals. We examined trends in RII and SII over time by including a two-way interaction term between the ridit-score and survey cycle (coded 1, 2, 3, 4, or 5) for each of the mental health outcomes. A statistically significant coefficient for the interaction term would indicate an upward or downward trend in the SII or RII. *P*-values < 0.05 (two tailed) were considered significant. All analyses were stratified by gender. In addition, supplementary analyses exploring potential gender differences for SIIs and RIIs were performed and are presented in Supplementary Table S1. Gender differences in RII and SII at each survey were specified by the inclusion of a two-way interaction term between the ridit-score and gender in each survey. Data management and statistical analyses was carried out using STATA version 13.

## Results

### Sample Characteristics

Descriptive statistics are shown in Table 1. Mean family affluence was stable (1.87–1.99) during the study period. More first-year secondary school students (47.4%) completed the questionnaire than second- (32.8%) and third-year students (19.8%). Respondents from Oslo, the capital of Norway, were included in 2015 and 2018. Table 2 shows that mean psychological distress, depressive, and anxiety symptoms as well as loneliness increased among girls and boys in all family affluence groups (not shown in table) during the study period. The prevalence of secondary school students with high symptom loads in all four mental health domains showed similar patterns.

**TABLE 1:** Unadjusted sample characteristics (*n* = 136,525) by survey year.

	2014 (*n* = 14,518)	2015 (*n* = 27,990)	2016 (*n* = 24,495)	2017 (*n* = 39,482)	2018 (*n* = 30,040)
**Gender**					
Boys	7,301(50.3)	13,894(49.6)	12,245(50.0)	19,608(49.7)	14,715(49.0)
Girls	7,217(49.7)	14,096(50.4)	12,250(50.0)	19,874(50.3)	15,325(51.0)
**School year**					
First year	6,629(45.7)	13,026(46.5)	14,152(57.8)	18,389(46.6)	12,189(40.6)
Second year	4,62(31.8)	8,909(31.8)	8,302(33.9)	12,746(32.3)	10,234(34.1)
Third year	3,269(22.5)	6,055(21.6)	2,041(8.3)	8,347(21.1)	7,617(25.4)
Living in Oslo	0	10,482(37.5)	0	0	10,768(35.9)
% high mental health loads*					
Psychological symptoms^a^	5,388(40.4)	11,062(43.7)	9,932(43.7)	18,559(49.9)	14,462(52.5)
Depressive symptoms^b^	6,976(51.8)	14,167(54.9)	12,522(54.7)	21,834(58.3)	16,761(60.3)
Anxiety symptoms^c^	2,556(19.1)	5,915(23.0)	5,278(23.2)	9,979(26.9)	8,152(29.7)
Loneliness^d^	1,222(9.1)	2,405(9.4)	2,415(10.6)	4,031(10.8)	3,353(12.1)
Mean affluence	1.98 (0.61)	1.87 (0.65)	1.92 (0.57)	1.99 (0.59)	1.94 (0.64)
Mean psychological distress*					
Psychological symptoms^a^	1.82 (0.66)	1.87 (0.69)	1.88 (0.71)	1.94 (0.66)	1.98 (0.68)
Depressive symptoms^b^	2.07 (0.78)	2.11 (0.79)	2.12 (0.80)	2.18 (0.80)	2.23 (0.82)
Anxiety symptoms^c^	1.45 (0.63)	1.51 (0.67)	1.53 (0.69)	1.58 (0.58)	1.61 (0.61)
Loneliness^d^	1.81 (0.98)	1.84 (0.99)	1.88 (1.01)	1.91 (1.01)	1.95 (1.03)

Data are n (%) or mean (SD) * sample size is reduced. ^*a*^n = 126,389. ^*b*^n = 127,446. ^*c*^n = 126,442. ^*d*^n = 127,035.

**TABLE 2:** Average psychological distress, prevalence’s of high symptom load, absolute and relative inequalities in mental health* among girls between 2014 and 2018 (five cycles) in the Ungdata survey.

	Psychological distress (*n* = 65,304)	Depressive symptoms (*n* = 65,820)	Anxiety symptoms (*n* = 65,310)	Loneliness (*n* = 65,625)
**Average mental health**				
2014	2.04 (2.0–2.06)	2.32 (2.30–2.33)	1.64 (1.62–1.65)	2.03 (2.00–2.05)
2015	2.10 (2.09–2.11)	2.37 (2.35–2.38)	1.71 (1.70–1.72)	2.06 (2.04–2.08)
2016	2.15 (2.13–2.16)	2.41 (2.40–2.43)	1.75 (1.74–1.76)	2.11 (2.09–2.12)
2017	2.17 (2.16–2.18)	2.45 (2.44–2.46)	1.75 (1.74–1.76)	2.12 (2.10–2.13)
2018	2.20 (2.19–2.21)	2.47 (2.46–2.48)	1.79 (1.78–1.80)	2.14 (2.12–2.16)
*p*-value for trend	*p* < 0.001	*p* < 0.001	*p* < 0.001	*p* < 0.001
**Prevalence high symptom loads**				
2014	0.55 (0.53–0.56)	0.65 (0.64–0.66)	0.29 (0.28–0.30)	0.13 (0.12–0.13)
2015	0.58 (0.57–0.59)	0.68 (0.68–0.69)	0.34 (0.33–0.35)	0.13 (0.13–0.14)
2016	0.60 (0.59–0.61)	0.70 (0.69–0.71)	0.35 (0.34–0.36)	0.15 (0.14–0.15)
2017	0.65 (0.65–0.66)	0.72 (0.72–0.73)	0.39 (0.38–0.40)	0.14 (0.14–0.15)
2018	0.66 (0.65–0.67)	0.73 (0.72–0.73)	0.42 (0.41–0.42)	0.15 (0.15–0.16)
*p*-value for trend	*p* < 0.001	*p* < 0.001	*p* < 0.001	*p* < 0.001
**Slope index of inequality^a^**				
2014	0.03 (–0.01 to 0.07)	0.01 (–0.02 to 0.05)	0.08 (0.04–0.12)	0.09 (0.06–0.12)
2015	0.05 (0.02–0.08)	0.03 (0.00–0.06)	0.08 (0.06–0.11)	0.09 (0.07–0.11)
2016	0.08 (0.04–0.11)	0.04 (0.00–0.07)	0.08 (0.05–0.11)	0.08 (0.06–0.11)
2017	0.10 (0.08–0.12)	0.09 (0.07–0.11)	0.13 (0.10–0.15)	0.10 (0.08–0.12)
2018	0.04 (0.01–0.07)	0.03 (0.01–0.06)	0.08 (0.05–0.11)	0.09 (0.07–0.11)
*p*-value for trend	*p* = 0.268	*p* = 0.055	*p* = 0.240	*p* = 0.610
**Relative index of inequality^b^**				
2014	1.06 (0.98–1.14)	1.02 (0.96–1.09)	1.34 (1.17–1.52)	2.06 (1.65–2.57)
2015	1.09 (1.04–1.14)	1.04 (1.00–1.08)	1.26 (1.16–1.37)	2.08 (1.78–2.43)
2016	1.14 (1.08–1.20)	1.05 (1.01–1.10)	1.27 (1.16–1.39)	1.77 (1.50–2.08)
2017	1.16 (1.12–1.21)	1.13 (1.09–1.16)	1.39 (1.31–1.48)	2.06 (1.82–2.33)
2018	1.06 (1.02–1.11)	1.05 (1.01–1.08)	1.21 (1.13–1.29)	1.84 (1.61–2.10)
*p*-value for trend	*p* = 0.793	*p* = 0.119	*p* = 0.535	*p* = 0.513

Data are regression-based predicted mean (95% CI) * adjusted for differences in respondents’ school year and whether respondent lives in Oslo. ^*a*^Represents the risk difference for poor mental health between the least and most affluent family. ^*b*^Represents the odds ratio for poor mental health between the lowest and the highest ranked family.

### Psychological Distress

**FIGURE 1:**
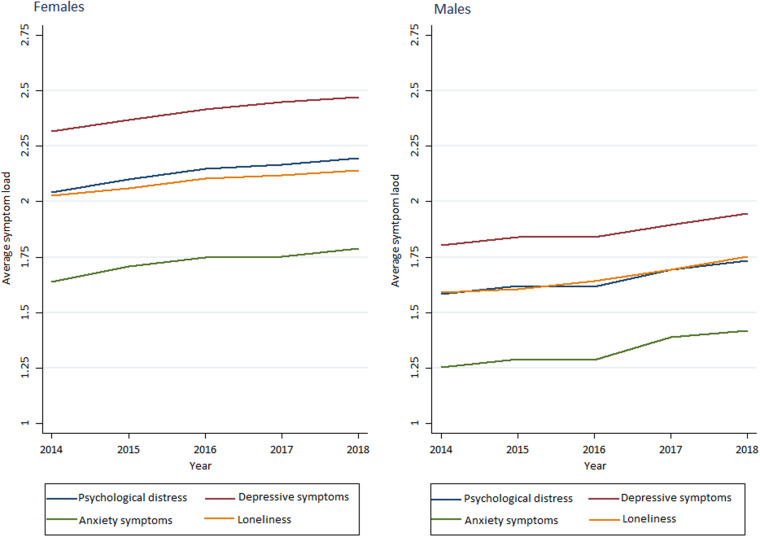
Trends in average mental health symptom loads among girls and boys across the five cross-sectional surveys of secondary school students (ungdata survey).

**FIGURE 2:**
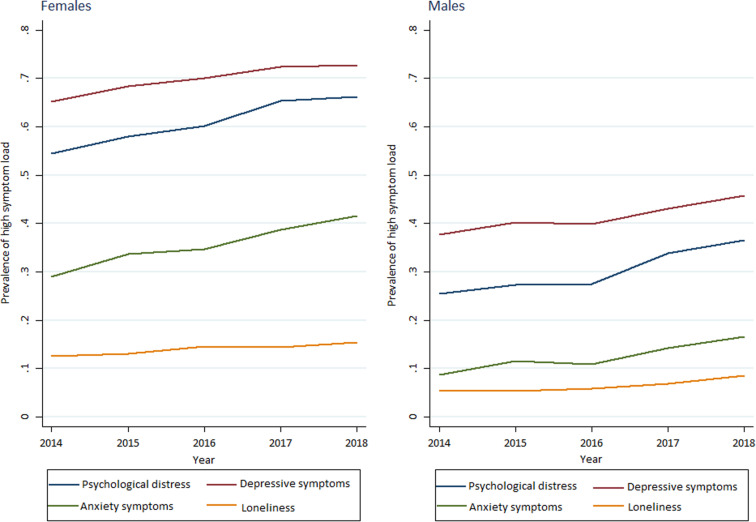
Trends in prevalence of high mental health symptom loads among girls and boys across the five cross-sectional surveys of secondary school students (ungdata survey).

As shown in Table 2 and summarized in Figures 1, 2, average scores of adolescents’ psychological distress increased both among girls (2.04–2.20, *p* < 0.001) and boys (1.58–1.73, *p* < 0.001) in the study period. Prevalence of adolescents with moderate to high symptom load also showed similar patterns (55–66%, *p* < 0.001 vs. 25–37%, *p* < 0.001 for girls and boys respectively). These trends were also significant after adjusting for family affluence, schoolyear, and whether respondent lives in Oslo.

Inequalities in mental health between affluence groups were observed in all five waves of the survey in both girls and boys (Tables 2, 3). While the overall absolute inequalities were stronger among boys than girls in 2018 (*p* = 0.005), the relative inequalities were stronger among girls in 2014 (*p* = 0.032), 2015 (*p* < 0.001), 2016 (*p* = 0.004), and 2018 (*p* < 0.001) (Supplementary Table S1).

**TABLE 3:** Average psychological distress, prevalence’s of high symptom load, absolute, and relative inequalities in mental health* among boys between 2014 and 2018 (five cycles) in the Ungdata survey.

	Psychological distress (*n* = 61,085)	Depressive symptoms (*n* = 61,626)	Anxiety symptoms (*n* = 61,132)	Loneliness (*n* = 61,410)
**Average mental health**				
2014	1.58 (1.57–1.60)	1.80 (1.79–1.82)	1.25 (1.24–1.26)	1.59 (1.57–1.61)
2015	1.62 (1.61–1.63)	1.84 (1.83–1.85)	1.29 (1.28–1.30)	1.61 (1.59–1.62)
2016	1.62 (1.61–1.63)	1.84 (1.83–1.85)	1.29 (1.28–1.29)	1.64 (1.62–1.66)
2017	1.69 (1.68–1.70)	1.90 (1.89–1.91)	1.39 (1.38–1.40)	1.69 (1.68–1.71)
2018	1.73 (1.72–1.74)	1.95 (1.93–1.96)	1.42 (1.41–1.43)	1.75 (1.73–1.77)
*p*-value for trend	*p* < 0.001	*p* < 0.001	*p* < 0.001	*p* < 0.001
**Prevalence high symptom loads**				
2014	0.25 (0.24–0.27)	0.38 (0.37–0.39)	0.09 (0.08–0.09)	0.05 (0.05–0.06)
2015	0.27 (0.27–0.28)	0.40 (0.39–0.41)	0.12 (0.11–0.12)	0.05 (0.05–0.06)
2016	0.28 (0.27–0.28)	0.40 (0.39–0.41)	0.11 (0.10–0.12)	0.06 (0.05–0.06)
2017	0.34 (0.33–0.35)	0.43 (0.42–0.44)	0.14 (0.14–0.15)	0.07 (0.07–0.07)
2018	0.37 (0.36–0.37)	0.46 (0.45–0.47)	0.17 (0.16–0.17)	0.09 (0.08–0.09)
*p*-value for trend	*p* < 0.001	*p* < 0.001	*p* < 0.001	*p* < 0.001
**Slope index of inequality^a^**				
2014	0.06 (0.02–0.10)	0.08 (0.04–0.12)	0.04 (0.02–0.06)	0.06 (0.04–0.08)
2015	0.08 (0.06–0.11)	0.07 (0.04–0.10)	0.06 (0.04–0.08)	0.05 (0.04–0.06)
2016	0.08 (0.05–0.11)	0.08 (0.04–0.11)	0.04 (0.02–0.06)	0.05 (0.04–0.07)
2017	0.07 (0.05–0.10)	0.06 (0.04–0.09)	0.06 (0.04–0.08)	0.05 (0.03–0.06)
2018	0.11 (0.08–0.14)	0.09 (0.06–0.12)	0.08 (0.05–0.10)	0.06 (0.04–0.07)
*p*-value for trend	*p* = 0.116	*p* = 0.407	*p* = 0.044	*p* = 0.940
**Relative index of inequality^b^**				
2014	1.27 (1.10–1.47)	1.24 (1.11–1.38)	1.56 (1.19–2.06)	3.14 (2.19–4.50)
2015	1.37 (1.24–1.52)	1.19 (1.10–1.28)	1.81 (1.53–2.15)	2.89 (2.21–3.77)
2016	1.38 (1.22–1.55)	1.24 (1.13–1.35)	1.50 (1.22–1.84)	2.61 (1.96–3.49)
2017	1.23 (1.15–1.33)	1.17 (1.10–1.24)	1.56 (1.37–1.77)	2.04 (1.68–2.47)
2018	1.33 (1.23–1.44)	1.22 (1.15–1.30)	1.60 (1.40–1.83)	1.93 (1.58–2.35)
*p*-value for trend	*p* = 1.000	*p* = 0.770	*p* = 0.696	*p* = 0.002

Data are regression-based predicted mean (95% CI) * adjusted for differences in respondents’ school year and whether respondent lives in Oslo. ^*a*^Represents the risk difference for poor mental health between the least and most affluent family. ^*b*^Represents the odds ratio for poor mental health between the lowest and the highest ranked family.

For girls, results suggest stable absolute (*p* = 0.268) and relative (*p* = 0.793) inequalities over the study period. Similar patterns were also observed in boys. Although the test for the linear trend in absolute and relative inequalities in psychological distress was not statistically significant in boys, SII increased from 0.06 (0.02–0.10) in 2014 to 0.11 (0.0–0.14) in 2018 and RII increased from 1.27 (1.10–1.47) in 2014 1 to 1.33 (1.23–1.44) in 2018.

### Depressive Symptoms

Mean depressive symptom scores increased during the study period in both girls (2.32–2.47, *p* < 0.001) and boys (1.80–1.95, *p* < 0.001). Prevalence of high depressive symptoms loads also increased in both genders (girls: 65–73%, *p* < 0.001 vs. boys: 38–46%, *p* < 0.001).

All surveys showed affluence inequalities among girls and boys (Tables 2, 3). Supplementary Table S1 shows that while absolute inequalities were stronger in boys than girls in 2014 (*p* = 0.022) and 2018 (*p* = 0.008), the relative inequalities were stronger in girls in 2014 (*p* = 0.002), 2015 (*p* = 0.006), 2016 (*p* = 0.002), and 2018 (*p* < 0.001).

Relative and absolute inequalities were stable over time in both genders. In girls, SIIs (0.01–0.03, *p* = 0.055) and RII (1.02–1.05, *p* = 0.119) slightly increased. However, the overall test for trend was not statistically significant in either RII or SII.

### Anxiety Symptoms

Anxiety symptom scores increased from 2014 to 2018 in both genders (girls; 1.64–1.79, *p* < 0.001 vs. boys; 1.25–1.42, *p* < 0.001). Similarly, prevalence of adolescents with high anxiety symptoms scores increased from 29 to 42% (*p* < 0.001) in girls and from 9 to 17% (*p* < 0.001) among boys.

Inequalities between affluence groups were evident in both genders. Supplementary analysis suggests that the overall absolute inequalities were stronger in girls than boys in 2016 and 2017. Similarly, the relative inequalities were larger in girls in 2015 (*p* < 0.001) and 2018 (*p* < 0.001).

In girls, results suggest stable relative and absolute inequalities. Among boys, the absolute inequalities slightly increased. In 2014, the most and least affluent family group differed by 0.04 in the proportion with high anxiety symptoms; by 2018 this difference had increased to 0.08 (*p* = 0.044). RII was stable during the same time period.

### Loneliness

Average symptoms of loneliness increased from 2014 to 2018 both among girls (2.03–2.14, *p* < 0.001) and boys (1.59–1.75, *p* < 0.001). The prevalence of high loneliness symptoms among boys increased from 5 to 9% (*p* < 0.001) and from 13 to 15% (*p* < 0.001) among girls. Thus, high loneliness is more prevalent among girls but growing much more quickly among males.

Absolute and relative inequalities were evident in both genders. Absolute inequalities were stronger among girls than boys in 2015 (*p* < 0.001), 2017 (*p* < 0.001), and 2018 (*p* = 0.004). Similarly, in 2014 (*p* = 0.051), 2015 (*p* = 0.040), and 2016 (*p* = 0.020) the RIIs was stronger in girls compared with boys (Supplementary Table S1).

In girls, results suggest stable absolute inequalities (*p* = 0.940), while relative inequalities were narrowing (3.14–1.93, *p* = 0.002) over the study period. Both absolute and relative inequalities were stable over time among girls.

## Discussion

The findings of this national representative study of five repeated cross-sectional surveys during the period 2014–2018 are consistent with our first hypothesis and with previous findings showing that psychological distress (in terms of symptoms of depression, anxiety, and loneliness) among adolescents are increasing over time (von Soest and Wichstrøm, 2014; OECD, 2018b; Potrebny et al., 2019). As expected, and in line with previous findings (Albert, 2015; Riecher-Rössler, 2017; Keyes et al., 2019), we found higher symptom scores and prevalence’s of moderate to high symptom loads among girls than boys, and thus compatible with our second hypothesis. Inequalities between socioeconomic groups were clearly evident in both boys and girls, with higher symptom load/distress with decreasing SES levels. In girls, absolute and relative affluence inequalities were stable in all mental health outcomes during the study period. Similarly, in boys, absolute and relative inequalities of psychological distress and depressive symptoms were stable. As a consequence, we found no support for our third hypothesis of widening inequalities according to SES. However, in boys, our results suggest widening absolute inequalities in symptoms of anxiety over successive surveys. Further, while the absolute inequalities in loneliness was stable over time, we observed narrowing relative inequalities from 2014 to 2018.

### Increasing Psychological Symptom Load Among Upper Secondary School Students in Norway

Our findings of increasing levels of mental health symptoms including depression, anxiety, and loneliness, with higher symptoms complaints among girls, is consistent with findings from other studies and national health reports (OECD, 2018b; Potrebny et al., 2019). These findings raise the question of why the recent generation of adolescents, and especially girls, report higher mental health symptoms load and greater risk of mental health complaints than previous cohorts. Increasing mental health complaints among the present population of adolescents in high-income western countries contrasts with improvements of their childhood conditions and increased material standards during the last decades. However, materialism and individualism in contemporary western societies may be hazardous for mental health (Eckersley, 2005). There is also a need to understand current time trends of adolescents’ mental health and other notable societal changes that have occurred during the twenty-first century (Gunnell et al., 2018). Although it is beyond the aim and scope of this paper, and we do not have suitable data to explore explanatory factors underlying these time trends, we will highlight some possible issues or hypotheses that subsequent studies might explore.

Several societal changes have occurred over the past twenty years that could adversely affect the mental health and contribute to a sustained increase in psychological distress in current generations of adolescents. Many high-income countries have, for example, experienced increasing wealth and expanded income inequality (OECD, 2019), both of which have been associated with adverse effects on adolescents’ mental health (Pickett and Wilkinson, 2007; Viner et al., 2012; Elgar et al., 2015). Moreover, changes in the family context in which children grow up undoubtedly affects children and adolescents well-being and mental health, such as increasing numbers of single-parent and step families (Barrett and Turner, 2005; Aasen Nilsen et al., 2018), parents with mental health and drug problems (Amone-P’Olak et al., 2011), and the emergence of overinvolved parents and parenting styles that place a lower value on children’s obedience (Twenge and Campbell, 2009; Schiffrin and Liss, 2017; Schiffrin et al., 2019). According to a Norwegian population study (Aasen Nilsen et al., 2018), adolescents living in single-parent and step families following divorce displayed higher adjustment problems than their peers living in non-divorced families. Overall, the groups scored higher on externalizing problems than on internalizing problems. Increasing rates of dual-earner households and children looked after in day-care facilities are also the norm in contemporary two-parent families (Green and Parker, 2006). Moreover, along with general changes in the family and the socioeconomic environment, there have also been several other changes in young people’s lifestyle, experiences, and expectations. These changes involve, among other things, increasing screen time and use of modern online technologies such as the internet, social media and online gaming (Hussain et al., 2017; Nesi et al., 2017; Twenge et al., 2019). Such online communities may be particularly attractive for lonely adolescents and those who are dissatisfied with their offline social relationships. Although the internet, social media, and online gaming offer numerous positive opportunities for connection, an increasing number of incidents have involved excessive/pathological use that may cause stress and lead to behavioral addiction, increased non-directional social comparisons and loneliness, low self-esteem, low social competence, low life satisfaction, and sleep disturbance as well as depressive and anxiety symptoms (Levenson et al., 2016; Rosenthal et al., 2016; Shensa et al., 2017; Baccarella et al., 2018; Twenge et al., 2018; Hawi and Samaha, 2019). A study by Nisar et al. (2019) found a positive correlation between passive Facebook use and increased non-directional social comparisons, which in turn was associated with depressive symptoms among the users. Moreover, online bullying is increasing, and several studies demonstrate correlations with symptoms of anxiety and depression and suicidal ideation in adolescents (Bauman et al., 2013; Patchin and Hinduja, 2017). Screen time and media use are also adversely associated with sleep health, primarily via delayed bedtime and reduced sleep duration (LeBourgeois et al., 2017). A significant share of school-aged children and adolescents get an insufficient amount of sleep (Owens and Group, 2014), which in turn is associated with worsened psychosocial health in adolescents (Tsuno et al., 2005; Shochat et al., 2014; Zhang et al., 2017).

There is no doubt that today’s generation of youths grows up in a time where the Internet and social media offer other opportunities for self-presentation than before, and there is a clear expectation to perform well in many life areas at once – such as in school and education, friendship and leisure activities, and body and appearance. A Norwegian study suggests that it is in particular the young people’s school situation that is most closely linked to young people’s stress-related mental health problems (Eriksen et al., 2017; Bakken et al., 2018; Bakken, 2019). Most adolescents cope well with this pressure, but for some it may become difficult to manage and master the totality of the demands and expectations they face. Perceiving oneself as having low social rank compared to others is, for example, demonstrated to be consistently linked to higher depressive symptoms (Wetherall et al., 2019). The evolutionary based social rank theory accounts for the inferiority and submissiveness that is typical of depression and social anxiety and understanding individuals’ perception of their social rank may thus enhance our understanding of depression etiology (Gilbert, 2019; Wetherall et al., 2019). Taken together, all of these changes may have a notable impact on the children, their parents’ practices, and the quality of parent-child relationships, which in turn influence children’s cognitive and socio-emotional development. On the other hand, increasing mental health symptom reports may be related to increased readiness to report symptoms. Changing attitudes about and awareness of mental health problems during the last decade may, thus, have contributed to increase frankness of reporting mental health symptoms (Collishaw, 2015).

One thing that has not changed is that girls and women are at higher risk of depression and anxiety disorders than boys and men, although these gender differences tend to increase with age (Potrebny et al., 2019). Researchers have proposed a number of explanations for this gender gap among adolescents. For example, some scientists have highlighted the “good girl syndrome” – with high expectations in many key areas of life such as in school, sports, leisure activities etc. – as an important factor in explaining the apparent gender differences in adolescent mental health (West and Sweeting, 2003; Wiklund et al., 2012). Recent national statistics show that more girls than boys experience high pressure in their daily lives; 16% of girls but only 6% of boys report that they often experience so much pressure that they have difficulties coping with it (Bakken, 2019). School pressure is the most prevalent type, and a slightly higher proportion of high SES adolescents experience high levels of pressure than their low SES peers (Bakken, 2019). A Swedish cross-sectional study of 16–18 years olds found that two-thirds of girls, but only one-third of boys, experienced significant school pressure (Wiklund et al., 2012). West and Sweeting (2003) suggest adolescent girls suffer an accumulation of worries about success in education and personal issues such as weight and appearance that combine to create elevated levels of stress, which adversely affect their mental health. Hankin et al. (2008) indicate girls are more socio-emotionally attentive than boys, and that negative cognitive style and rumination may interact to predispose girls to mental health complaints, particularly depressive symptoms.

### Stable, but Significant Socioeconomic Differences in Adolescent Mental Health in Norway During 2014–2018

In line with previous findings (Bøe et al., 2012, 2014; Zhou et al., 2018), we found socioeconomic inequalities in all four mental health outcomes. A decrease in SES was associated with increasing average scores and prevalence’s of moderate to high symptom load of psychological distress, depression and anxiety, as well as loneliness. Previous studies and international reports suggest that the mental health gap between advantaged and disadvantaged children and youth has not reduced over the last decades and some even suggest widening inequalities (Inchley and Currie, 2013; Elgar et al., 2015). Elgar et al. (2015) explored trends in socioeconomic inequalities in adolescent’s health across 34 North American and European countries, suggesting increasing inequalities in physical activity, body mass index, and psychological and physical health symptoms. Our results, however, did not find proof for narrowing or increasing inequalities, in either absolute or relative terms, in either boys or girls during the study period. These findings are unsurprising due to the relatively short follow-up time in the present study (5 years). Exploring time trends require assessing change over sufficiently long time periods and according to Eimecke et al. (2011) periods exceeding 7 years are necessary to identify secular change.

Although these findings align with an enormous amount of literature, the explanations for these stark differences are far from conclusive. As described initially, factors associated with the parents and family environment are, along with biological factors, the most important mediating variables between SES and young people’s mental health. Within this area of research, the family process and family investment model are considered as most dominant. The family process model assumes a link between family socioeconomic status and children’s socioemotional development and adjustment through the psychological wellbeing of parents and thereby their childrearing practices (Conger et al., 1994; Conger and Conger, 2002). Similarly, a Norwegian population study by Bøe et al. (2014) found a higher prevalence of parents with mental health complaints in low-income families and these parents used in turn more negative upbringing strategies (such as penalties and scolding). Negative upbringing strategies were also more prevalent among low educated parents. The family investment perspective is based on more materialistic explanations and parents’ ability to spend time with children and to invest in leisure activities, books and learning materials, stable and comfortable housing conditions, etc. (Knitzer and Perry, 2009; Mayer, 2010). According to this perspective, which most proponents use to explain socioeconomic disparities in children’s cognitive development and educational attainment, restricted access to resources that contribute to positive development affects the development of children in low-income families (Knitzer and Perry, 2009). There are, however, many indications that family process and family investment influence each other and that a more complete understanding of the relationships between SES and family life should combine these two models (Conger et al., 2010). Studies by Linver et al. (2002) and Yeung et al. (2002) examined predictors from the family process model and the family or parental investment perspective and each study found that family stress processes were better predictors of behavioral problems, whereas family investment were better predictors of cognitive development. Yeung et al. (2002) comprehensive study included a survey of 735 children 3–5 years old, collecting information on family investment and family processes as well as comprehensive information on children’s cognitive and socio-emotional development. Their results indicated that the more structural components from the family investment perspective and the more affective components from the family process model were both central to shaping the complex relationship between low income and children’s cognitive and socio-emotional development. The study also demonstrated mutual influence between the mechanisms described in the two different perspectives: a home environment with a higher material standard was more cognitively stimulating and positive for children’s learning. However, this environment was also positive for maternal mental health and mothers’ use of positive parenting strategies, which in turn was positive for children’s socio-emotional development.

Most adolescents spend a significant amount of their time outside the family environment and are undoubtedly also influenced by these more distal environments such as peers, neighborhood, school, leisure activities, social media, etc. The family’s affluence level affects, to a large extent, the school environment and the neighborhood of residence of the children (Evans et al., 2012). Research also demonstrate differences in adolescents’ health behavior and living habits across different affluence levels (Marmot and Bell, 2012; Viner et al., 2012). Low family SES is, for example, unfavorably associated with physical activity, sport participation, and screen-based behaviors in adolescents (Langlois et al., 2017; Mielke et al., 2017). Previous studies suggest inequalities in sleep health and that adolescents from low income families may be at even greater risk of poor-quality and insufficient sleep (Marco et al., 2012; Owens and Group, 2014; Sivertsen et al., 2017). Moreover, according to the relative deprivation theory it may be detrimental for individuals’ mental health to be unable to afford goods or activities that are considered affordable to most (Sweet, 2011; Adjaye-Gbewonyo and Kawachi, 2012). This would particularly be true for adolescents given their strong tendency to value and conform to peer norms. Future research should take this into account when exploring time trends in socioeconomic inequalities in health-related outcomes.

### Strengths and Limitations of the Study

A main strength of the study is its large, nationally representative samples, which allowed us to explore trends in adolescents’ mental health on a large scale. This study is based on five comparable and well-designed surveys conducted each year during 2014–2018 in several Norwegian upper secondary schools. Moreover, the family affluence level and adolescents’ symptoms of depression were measured in a standardized manner by using validated measures (Derogatis et al., 1974; Strand et al., 2003; Currie et al., 2008).

Some limitations of this study should be noted. First, although overall response rates were relatively high, about 30% of students in the surveyed schools were absent from the surveys, which poses the possibility of non-response bias due to illness and truancy, both of which likely interact with our variables of interest. Second, the schools and municipalities included in the study sample differ in each survey/study year. The socio-demographic distribution of the population of Norwegian municipalities varies and it is therefore important to take this into account when interpreting the results of this study. All analyses are adjusted for whether the respondent live in Oslo, the capital of Norway. In addition, we performed the parametric estimation without respondents from Oslo (results not shown), and results did not change significantly. Third, our outcome variables were self-reported, introducing a risk of misclassification and measurement bias. Moreover, the reliability of the anxiety and loneliness measures is uncertain and use of exclusively validated instruments would have strengthened the study findings. Fourth and finally, exploring time trends requires assessing change over sufficiently long time periods. Eimecke et al. (2011) suggest that periods exceeding 7 years are necessary to identify secular change. The relatively short follow-up time in the present study (5 years) thus reduces our ability to detect potential changes related to socioeconomic inequalities in adolescents’ mental health over time. Despite these caveats, these results still have some implications for the public health of adolescents in Norway as well as for other (comparable) high-income western countries.

### Implications

Rising rates of psychological distress among adolescents have long-term consequences in terms of individual education attainment and occupational and socioeconomic outcomes, health and quality of life in adulthood, and relationships and parenting for the next generation. Taken together, rising mental health complaints in the youth population may have formidable long-term consequences for the individuals involved and for society at large in terms of increased economic costs, reduced productivity, and increased socioeconomic inequalities. Health inequalities among adolescents shape future inequalities in education, employment, health, and quality of life in adulthood and socioeconomic circumstances in future generations.

The strong links shown in our study between long-term consequences, inequalities, and mental health demonstrate a need for evidence-based interventions to mitigate these links. Our study results penetrate multiple levels of society such as family, housing, and neighborhood conditions. Thus, multilevel interventions and strategies across different sectors of society are required. Interventions may reduce mental health inequalities in different contexts, many of which lie outside of health services. There are effective early interventions that can promote mental health in vulnerable groups, but our results underline the necessity of both initiating and facilitating a cross-sectoral approach and of forming partnerships between different government departments, civic society organizations, and other relevant stakeholders. The growing body of research on effective cross-sectoral interventions addressing “upstream factors” such as parenting, housing, and neighborhood conditions is promising. The EU Member States and the European Commission have published a report recommending the Mental Health in all Policies (MHiAP) approach to promote population mental health (Eu Directorate General for Health and Food Safety, 2015), arguing for a life course approach to poverty and mental health with interventions targeting specific age groups (Wahlbeck et al., 2017). This MHiAP approach can be applied to all public policy levels, from local to international. Hence, our findings not only underline the need to tackle social inequalities; they also emphasize the necessity of applying frameworks such as the MHiAP to stitch together what has traditionally been fragmented and intermittent.

## Conclusion

In conclusion, our results show a trend of increasing psychological distress among upper secondary school students, across all socioeconomic groups and in particular among girls since 2014. Inequalities in psychological distress according to socioeconomic status (SES) remained stable through this period. As well as being unjust, this represents a serious public health problem. These findings suggest that current strategies have not been sufficient in addressing adolescents’ mental health challenges and inequalities, thereby revealing the need to intensify efforts in social and health policy, public health, and further research. Since existing studies point toward the positive mental health effects of many non-health policies, such as social, labor market, and housing policies, implementation of effective interventions in a MHiAP framework should be exaggerated by adding resources in non-health fields to the common goal of improving wellbeing for all.

## Data Availability Statement

Norwegian Social research (NOVA) administers and maintains the Ungdata database. Data is freely available for research and educational purposes from the Norwegian centre for research data (NSD) upon application. Details about the application process can be found at: https://nsd.no/nsddata/serier/ungdata_eng.html. Norwegian legislation prohibits deposition of these data to open archives.

## Ethics Statement

The studies involving human participants were reviewed and approved by the Norwegian Centre for Research Data (NSD), https://nsd.no/nsd/english/index.html. Informed consent to participate in this study was obtained from the students, as well as from legal guardian/next of kin if the student was under 16 years of age.

## Author Contributions

AM, ES, ML, and KA contributed substantially to the conceptualization and design of the study. AM performed and had primary responsibility for all data management, statistical analysis, interpretation of the results, and writing the manuscript. ES assisted with the data management, statistical analysis, interpretation of results, and editing of the manuscript. ML and KA assisted with writing and editing of the manuscript. All authors read and approved the final manuscript and take responsibility for the integrity of the data analysis and the decision to submit this manuscript for publication.

## Conflict of Interest

The authors declare that the research was conducted in the absence of any commercial or financial relationships that could be construed as a potential conflict of interest.
