# Supplementary material for: Trends in Socioeconomic Inequalities in Norwegian Adolescents’ Mental Health From 2014 to 2018: A Repeated Cross-Sectional Study
Source: Front Psychol. 2020 Jul 7;11:1472. doi: 10.3389/fpsyg.2020.01472 (PMC7358281; doi:10.3389/fpsyg.2020.01472)
Supplement: Supplementary file 1 [file Table_1.pdf]

Supplementary Material

Supplementary table 1: Absolute and relative inequalities in mental health and their interaction with gender* for each survey in 2014 to 2018 in the Ungdata survey

	2014	2015	2016	2017	2018
			**Phycological distress**		
Absolute inequalities					
Female	0.06 (0.02-0.10)	0.08 (0.06-0.11)	0.08 (0.05-0.11)	0.07 (0.05-0.10)	0.10 (0.07-0.13)
SII	0.32 (0.28-0.35)	0.34 (0.31-0.36)	0.33 (0.30-0.36)	0.32 (0.30-0.34)	0.32 (0.30-0.35)
Female; SII	-0.03 (-0.08-0.03)	-0.03 (-0.07-0.01)	0.00 (-0.05-0.04)	0.03 (-0.01-0.06)	-0.06 (-0.10 to -0.02)
Relative inequalities					
Female	1.27 (1.10-1.47)	1.35 (1.22-1.50)	1.38 (1.22-1.55)	1.23 (1.15-1.33)	1.30 (1.20-1.40)
RII	2.50 (2.24-2.79)	2.51 (2.32-2.71)	2.54 (2.33-2.76)	2.10 (1.99-2.22)	2.03 (1.90-2.15)
Female; RII	0.84 (0.71-0.98)	0.81 (0.72-0.90)	0.83 (0.72-0.94)	0.94 (0.87-1.02)	0.82 (0.76-0.90)
			**Depressive symptoms**		
Absolute inequalities					
Female	0.08 (0.04-0.12)	0.07 (0.04-0.10)	0.08 (0.04-0.11)	0.06 (0.04-0.09)	0.09 (0.06-0.12)
SII	0.32 (0.28-0.36)	0.31 (0.28-0.33)	0.33 (0.30-0.35)	0.29 (0.27-0.32)	0.29 (0.27-0.32)
Female; SII	-0.07 (-0.12 to -0.01)	-0.04 (-0.08-0.00)	-0.04 (-0.09-0.00)	0.02 (-0.01-0.06)	-0.05 (-0.09 to-0.01)
Relative inequalities					
Female	1.24 (1.11-1.38)	1.17 (1.09-1.26)	1.24 (1.13-1.35)	1.17 (1.10-1.24)	1.20 (1.13-1.28)
RII	1.98 (1.82-2.15)	1.85 (1.75-1.96)	1.97 (1.85-2.10)	1.78 (1.70-1.86)	1.72 (1.63-1.81)

Female; RII	0.82 (0.73-0.93)	0.89 (0.82-0.97)	0.85 (0.77-0.94)	0.96 (0.90-1.03)	0.87 (0.81-0.94)
**Anxiety symptoms**					
Absolute inequalities					
Female	0.04 (0.02-0.06)	0.06 (0.04-0.08)	0.04 (0.02-0.06)	0.06 (0.04-0.08)	0.08 (0.05-0.10)
SII	0.20 (0.17-0.23)	0.23 (0.21-0.25)	0.23 (0.20-0.25)	0.24 (0.22-0.25)	0.26 (0.23-0.28)
Female; SII	0.04 (0.00-0.09)	0.02 (-0.02-0.05)	0.04 (0.00-0.08)	0.07 (0.04-0.10)	0.00 (-0.03-0.04)
Relative inequalities					
Female	1.56 (1.19-2.06)	1.78 (1.50-2.11)	1.50 (1.22-1.84)	1.56 (1.37-1.77)	1.55 (1.36-1.77)
RII	3.82 (3.12-4.66)	3.76 (3.30-4.30)	3.69 (3.19-4.26)	3.10 (2.82-3.41)	2.97 (2.68-3.30)
Female; RII	0.85 (0.63-1.16)	0.71 (0.59-0.86)	0.85 (0.68-1.06)	0.89 (0.77-1.03)	0.78 (0.67-0.91)
**Loneliness**					
Absolute inequalities					
Female	0.06 (0.04-0.08)	0.05 (0.04-0.06)	0.05 (0.04-0.07)	0.05 (0.03-0.06)	0.05 (0.04-0.07)
SII	0.07 (0.05-0.09)	0.07 (0.05-0.08)	0.08 (0.07-0.10)	0.06 (0.05-0.08)	0.06 (0.04-0.08)
Female; SII	0.03 (0.00-0.06)	0.04 (0.02-0.06)	0.03 (0.00-0.06)	0.05 (0.03-0.07)	0.04 (0.01-0.06)
Relative inequalities					
Female	3.14 (2.19-4.50)	2.88 (2.21-3.77)	2.61 (1.96-3.49)	2.04 (1.68-2.47)	1.90 (1.56-2.31)
RII	3.33 (2.47-4.48)	3.17 (2.54-3.95)	3.28 (2.63-4.09)	2.22 (1.90-2.58)	1.93 (1.64-2.29)
Female; RII	0.66 (0.43-1.00)	0.72 (0.53-0.99)	0.68 (0.49-0.94)	1.01 (0.80-1.27)	0.97 (0.77-1.23)

Data are regression-based predicted mean (95%CI) *adjusted for differences in respondents' school year and whether respondent lives in Oslo [a] Represents the risk difference for poor mental health between the least and most affluent family. [b] Represents the odds ratio for poor mental health between the lowest and the highest ranked family
